# Clinical risk factors for late intestinal toxicity after radiotherapy: a systematic review protocol

**DOI:** 10.1186/2046-4053-2-39

**Published:** 2013-06-07

**Authors:** Qiyuan Qin, Qingshan Huang, Qinghua Zhong, Xinjuan Fan, Dianke Chen, Lei Wang

**Affiliations:** 1 Department of Colorectal Surgery, Sixth Affiliated Hospital, Sun Yat-sen University, 26 Yuancun Er Heng Road, Guangzhou, 510655 Guangdong, P.R. China; 2 Medical Library, Sun Yat-sen University, 74 Zhongshan Er Road, Guangzhou, Guangdong, 510080, P.R. China; 3 Department of Pathology, Sixth Affiliated Hospital, Sun Yat-sen University, 26 Yuancun Er Heng Road, Guangzhou, Guangdong, 510655, P.R. China; 4 Department of Oncology, Sixth Affiliated Hospital, Sun Yat-sen University, 26 Yuancun Er Heng Road, Guangzhou, Guangdong, 510655, P.R. China

**Keywords:** Intestinal toxicity, Radiation injury, Radiotherapy adverse effect, Risk factor, Chronic radiation enteritis, Systematic review

## Abstract

**Background:**

Late intestinal toxicity after radiotherapy (LITAR) not only limits the radiation dose, which subsequently leads to unfavorable clinical outcomes, but also significantly lowers the quality of life in an increasing number of cancer survivors. Therefore, identifying clinical risk factors for LITAR is important for establishing a predictive model in the clinical setting of decision-making for these patients. This review aims to systematically summarize and clarify the clinical factors that can be potentially associated with an increased risk of moderate/severe LITAR in patients with abdominal or pelvic malignancies.

**Methods/design:**

MEDLINE, EMBASE, Web of Science, Cochrane Central Register of Controlled Trials, Scopus, Google Scholar and Chinese BioMed will be systematically searched to identify appropriate studies. Citations of the retrieved studies and recent reviews will also be searched separately by case.

The enrolled studies should at least have the following information: (1) a clear definition and information on the LITAR severity; (2) assess clinical factors for moderate/severe toxicity with adjusted risk estimates; (3) have a cohort, case–control, randomized controlled trial and controlled clinical trial study design.

Two authors will independently review the abstract and full text of retrieved studies, extract data from eligible studies and assess the risk of bias. Disagreements will be discussed among reviewers until a consensus is reached. The effect of identified risk factors will be displayed in forest plots. If the information is sufficient, results will be synthesized by a meta-analysis with the random effects model to pool the estimate of risk posed by clinical factors. Subgroup and sensitivity analysis will be used to explore the sources of heterogeneity.

**Discussion:**

This review will summarize the evidence of clinical risk factors for moderate/severe LITAR. The results may help guide decision-making and minimize the side effects of therapeutic modalities in the clinical setting.

**Trial registration:**

This review has been registered at PROSPERO. The registration no. is CRD42013003744.

## Background

Radiotherapy is one of the most effective management methods for solid malignancies. It is estimated that about 50% of all cancer patients will receive radiotherapy in the course of treatment for the disease [1]. Data from the International Agency for Research on Cancer (IARC) indicates that there are about 12.7 million new cancer cases per year worldwide. Among those patients, more than 5.2 million suffer from either abdominal or pelvic malignancies [2,3]. Studies have showed that 14-68% of patients with abdominal or pelvic cancers eventually undergo radiotherapy for either curative or palliative care [1]. There is no doubt that radiotherapy accompanied by other therapeutic modalities has greatly contributed to increased long-term survival rates for these patients, although treatment-associated toxicities are commonly found [4-6].

Late intestinal toxicity after radiotherapy (LITAR) for abdominal or pelvic cancers, resulting from exposure of the nontarget bowel area and irreversible progression of lesions, could not be averted even by the most advanced radiation techniques [6-8]. LITAR, sometimes called chronic radiation enteritis (CRE), is widely defined as major intestinal toxicities or complications occurring or persisting more than 3 months after radiotherapy. Potential symptoms include significant diarrhea, constipation, distention, abdominal or bottom pain, rectal bleeding and complications of fecal incontinence, bowel obstruction and fistulas [7-10]. The prevalence of LITAR varies across studies depending on the grading criteria, time of follow-up and study design. Of note, about 80-90% of patients who have undergone abdominal or pelvic radiotherapy will develop a permanent change in bowel habits, and the quality of life of half of these patients will be affected [10-12]. Despite the high incidence of mild symptoms, it is commonly recognized that the incidence of moderate/severe LITAR is 5.6-15% [13-17].

Moderate/severe LITAR not only limits the radiation dosage and target effect, but also significantly lowers the quality of life of cancer survivors [5,18]. It is essential to strike a balance between the therapeutic benefits and side effects of radiotherapy. Unfortunately, the early symptoms of LITAR, without enough specificity, are often masked by drug effects or other gastrointestinal diseases [7,19]. Moreover, the separate patterns of modern cancer therapy prevent doctors in different fields from having an overview of patients’ conditions, further obscuring the diagnosis and treatment strategy for LITAR [7,9]. Therefore, it is imperative to identify risk factors for developing moderate/severe LITAR that could be pivotal in recognizing potential patient populations that are more likely to have side effects versus who are not prior to the administration of radiotherapy.

Previous studies have focused on investigating radiation dosimetric factors that play a direct role in the development of intestinal injuries, aiming to establish a predictive model of Normal Tissue Complication Probability (NTCP) with radiation parameters [20-23]. Genetic profiling is another promising tool for risk stratification of these patients, which is supported by that fact that some studies have verified gene markers for patients who are predisposed to radiotherapy-associated side effects [5,24-26]. However, these findings need further confirmation by independent studies and multicenter clinical trials [5,27,28].

On the other hand, clinical characteristics might be alternative factors that can predict the outcomes of treatments. Clinical factors, such as general physical condition, lifestyle, accompanying diseases, and relevant therapy, among others, account for much of the variation in normal tissue reactions to radiation and affect the outcomes of patients after radiotherapy [7,29]. Consequently, developing a practical and useful prediction model using clinical risk factors for oncologists, radiotherapists, and gastroenterologists would be a significant contribution. Increasingly, studies are describing the clinical risk factors for moderate/severe LITAR [15,30-33]. Nevertheless, the findings from many studies are still controversial [7]. To date, no systematic review has summarized the clinical risk factors that are potentially associated with LITAR. Thus, in this study, we conducted a systematic review to summarize and clarify which clinical factors are associated with an increased risk of moderate/severe LITAR in patients who have undergone radiotherapy for pelvic or abdominal malignancies.

## Methods/design

We will follow the recommendations made by Preferred Reporting Items for Systematic Reviews and Meta-Analyses (PRISMA) [34] and the Meta-analysis Of Observational Studies in Epidemiology (MOOSE) group [35] throughout the course of this review. The PRISMA and MOOSE checklists are described in detail in Additional file 1. Ethics is not required given the protocol is for a systematic review.

This review has been registered at PROSPERO. The registration no. is CRD42013003744.

### Data sources

Electronic databases and clinical trials registries will be searched. MEDLINE, EMBASE, Web of Science, Cochrane Central Register of Controlled Trials (CENTRAL), Scopus, Google Scholar and Chinese BioMed (CBM) from 1990 to 2013 will be systematically searched. Since the well-accepted radiation toxicity criteria were initially published in the 1990s [36,37], studies from before that time are not comparable with others because of the different definition and grading system used. Moreover, multivariate analysis and analysis adjusted for confounding factors were seldom used before the 1990s, which could potentially cause an additional bias by chance. Therefore, we will focus on studies that were carried out after 1990. In addition, we will also look through the citations in the retrieved studies and recent reviews to avoid missing relevant studies in this field.

### Search strategy

In conjunction with a librarian (QH) and a researcher with experience in systematic reviews (RZ), we developed a systematic search strategy with structured terms of medical subject headings (MeSH) and free keywords. Three themes will be searched for, including “radiation toxicity,” “intestinal diseases and symptoms,” and “radiotherapy for abdominal or pelvic malignancy.” There is no restriction on the study language. The search strategy for MEDLINE in PubMed is described in detail in Additional file 2, which will be adjusted to search for eligible studies in other databases as well.

### Eligibility criteria

Studies will be included if they fulfilled the following criteria.

#### Population of interest

Included studies should target populations with primary diseases that require pelvic radiotherapy, including:

1. Genitourinary malignancies of males and females, such as prostate cancer, bladder cancer, cervical cancer, endometrial cancer, etc.;

2. Gastrointestinal malignancies, such as colorectal cancer, anal cancer, etc.;

3. Other malignant diseases that require abdominal or pelvic radiotherapy, such as non-Hodgkin’s lymphoma, etc.

Of note, studies only on animals will be excluded.

#### Risk factors of interest (intervention/exposure)

Included studies should assess at least one clinical risk factor other than radiation dosimetric variables. Clinical risk factors include patients’ demographics (race, ethnicity, age, BMI, education level, economic status), lifestyle (alcohol use, smoking), history of pre-radiation medical disorders and gastrointestinal symptoms, accompanying diseases (diabetes, hypertension, cardiovascular disease), concomitant treatments (chemotherapy, hormone therapy) and acute radiation-induced intestinal toxicity.

#### Comparators

Comparator groups of cohort and intervention studies should be set with the same primary diseases and comparable radiation plans, but without specific risk factors. For case–control studies, comparator groups should be populations that received radiotherapy for the same primary diseases but did not have moderate/severe LITAR.

#### Study outcomes

The primary outcome of this review is moderate/severe LITAR. Studies describing risk factors for LITAR without grading or only measuring mild symptoms will be excluded. In addition, only studies providing risk estimates adjusted for at least one confounding factor will be included.

Included studies should use a similar definition of LITAR, which is major intestinal toxicities or complications occurring or persisting more than 3 months after radiotherapy [7-10]. The severity of LITAR should be evaluated in accordance with the Radiation Therapy Oncology Group/European Organization for Research and Treatment of Cancer (RTOG/EORTC) scoring scale [36,38] or Common Terminology Criteria for Adverse Events (CTCAE) from the National Cancer Institute [39]. Self-reported symptoms should be assessed on the basis of Late Effects in Normal Tissues Subjective, Objective, Management and Analytic (LENT SOMA) scales, incorporated into CTCAE [37,40]. Grade 2 and higher according to RTOG/EORTC or LENT SOMA/CTCAE will be considered to be moderate/severe.

#### Study designs

Observational cohort and case–control studies, randomized controlled trials (RCTs) and controlled clinical trials (CCTs) for interventions will be included. Cross-sectional studies, case series, case reports, experimental studies and reviews will be excluded.

### Study selection

Two reviewers (QZ, ZY) will separately and independently screen the titles and abstracts of studies identified from initial searches. A standard screening checklist based on the eligibility criteria above will be employed for each study. Studies that do not meet the criteria according to the titles or abstracts will be excluded. Full-text versions of the remaining studies, including those that are potentially eligible studies and uncertain, will be retrieved for a second review by at least two reviewers (QZ, ZY, QQ) independently to determine the eligibility. Disagreements with regard to study eligibility will be further discussed among reviewers. If consensus cannot be reached, a third reviewer (LW) will make the ultimate decision.

For studies without sufficient information to evaluate the eligibility, we will contact the study authors via email to obtain their clarifications. The studies will be excluded if there is still insufficient information after this contact.

If more than one publication reports the results from the same study population, we will choose the publication with the largest sample size. The abstracts that were published in academic conferences will be evaluated by case, and we will contact the study authors for details if necessary. The abstracts or full texts of studies not published in English will be translated by Google Translate. If the information is unclear, we will refer to professional translations.

### Data extraction

We will develop three kinds of data extraction form corresponding to different study designs. There will be specific forms for cohort studies, case–control studies, and RCTs or CCTs. The data extraction form used for cohort studies is described in detail in Additional file 3.

The data items mainly abstracted are as follows: title, first author, year of publication, publication journal, funding source and conflict of interest, trial code designation, study design (prospective, retrospective), study characteristics (primary malignancy, endpoint definition, study period, sample size, control conditions, interventions/exposures, randomization, blinding), study population and patient demographics (gender ratio, patients age range, race and ethnicity), radiation instrument, radiotherapy plan (total dose, external beam radiation dose, brachytherapy dose, dose fractionation, target area arrangement) and results (follow-up period, number of patients lost to follow-up, number of cases, identified risk factors, adjusted effect estimates and 95% CI). We will extract or calculate the adjusted estimates of the odds ratio (OR), rate ratio (RR), hazard ratio (HR) for factors of interest and associated 95% CIs from eligible studies.

Two review authors (QZ, ZY) will separately and independently extract the data from the eligible studies. Disagreements regarding the data extraction between authors will be resolved by discussion. If consensus cannot be reached, a third author (LW) will review the study and arbitrate. If data are missing for synthesis or assessment of study quality, we will attempt to contact the study authors via email at least two times. The study will be excluded if there is still insufficient data following this process.

### Assessment of quality (risk of bias)

The study characteristics regarding the risk of bias will be extracted to evaluate study quality by two review authors (RZ, QQ) independently. Disagreement between authors in respect of study quality assessment will be resolved by discussion.

The assessment items for cohort studies, as outlined in the Newcastle-Ottawa scale [41], include cohort selection (representativeness of the exposed cohort and non exposed cohort, ascertainment and timing of the exposure), comparability and outcome measure (assessment method, time and adequacy of follow-up). The assessment items for case–control studies, as outlined in the Newcastle-Ottawa scale [41], include case selection (definition and representativeness of cases and controls), comparability and exposure measure (assessment method, parallel between cases and controls, response rate). The assessment items, outlined in the Cochrane Collaboration’s tool for assessing the risk of bias for RCTs [42], include the method of randomization, concealment of treatment allocation, blinding of patients and researchers throughout the trial (intervention, data collection and analysis), completeness of outcome data, selective report of outcomes and any other potential sources of bias. There is no special tool to assess the risk of bias for CCTs, which will be evaluated based on blinding, comparability, completeness of outcome data and selective report of outcomes.

The levels of assessment items will be displayed for each eligible study accompanying the published manuscript.

### Statistical analysis

#### Data synthesis and analysis

The effects of risk factors will be explored in forest plots. A meta-analysis will be applied to each factor if the definitions of outcomes and characteristics among studies are comparable. Because the incidence of moderate/severe LITAR is relatively low, we consider that the estimate of OR could approximate the estimate of relative risk (RR, HR). Subsequently, we will use OR as the effect measure for meta-analysis in the current review [43].

The effect of residual confounding factors is an unavoidable limitation of meta-analyses of observational studies. Therefore, we will use the random effects model of Dersimonian and Laird [44] to pool the overall adjusted OR and associated 95% CI, based on our assumption that there is potential variation among studies of different populations, designs, primary malignancies and radiation plans.

Different risk factors are confounders to each other. If the information is sufficient, the pooling estimate of one factor will be adjusted for other potential risk factors.

#### Assessment of heterogeneity

Heterogeneity across all the studies will be assessed by the Cochran Q test and *I*^*2*^ statistic. The latter could reflect the magnitude of between-study variation attributed to heterogeneity, which is preferred with an increased number of included studies [45].

Subgroup analysis will be further used to explore the sources of heterogeneity. When data are sufficient, subgroups will be stratified as follows: study population, study design (prospective *vs*. retrospective), regions of intestinal toxicity (colorectum *vs*. small intestine), primary malignancies and radiation instruments.

We will also perform sensitivity analysis to assess the robustness of results with variation in study quality, time of follow-up, effect measures (OR/ RR) and severity of LITAR.

#### Assessment of publication bias

The results will be displayed in forest plots and cumulative forest plots, which provide a visual and direct impression of effect size and publication bias. We will also perform Peters’ regression analysis to assess the trend with a funnel plot [46].

StataSE (version 11.0; StataCorp, College Station, TX) will be used for data synthesis, assessment of heterogeneity and publication bias.

## Discussion

This systematic review will summarize the evidence of clinical risk factors for moderate/severe late intestinal toxicity in patients who have undergone abdominal or pelvic radiotherapy. In summary, patients’ demographics, lifestyles, history of previous medical disorders, concurrent diseases and concomitant treatment will be taken into account. The enrolled studies for this review will include cohort studies, case–control studies, RCTs and CCTs. A meta-analysis will also be used to explore the estimate of risk factors if necessary.

The finding of this review will be widely disseminated through peer-reviewed publications, conference presentations and other health information press sources. This will provide comprehensive information that might be of importance with regard to individualized treatment of malignancies and will help to archive the early detection of major LITAR. In light of specific clinical risk factors, health care providers could inform and discuss with patients to make optimized clinical decisions, ultimately leading to averting unnecessary suffering and economic losses.

## Abbreviations

IARC: International agency for research on cancer; LITAR: Late intestinal toxicity after radiotherapy; CRE: Chronic radiation enteritis; NTCP: Normal tissue complication probability; PRISMA: Preferred reporting items for systematic reviews and meta-analyses; MOOSE: Meta-analysis of observational studies in epidemiology; CENTRAL: Cochrane central register of controlled trials; CBM: Chinese BioMed; RTOG/EORTC: Radiation Therapy Oncology Group/European Organization for Research and Treatment of Cancer; CTCAE: Common terminology criteria for adverse events; LENT SOMA: Late effects in normal tissues subjective objective, management and analytic; BMI: Body mass index; RCTs: Randomized controlled trials; CCTs: Controlled clinical trials; OR: Odds ratio; RR: Rate ratio; HR: Hazard ratio.

## Competing interests

Author Qiyuan Qin and Lei Wang are carrying out a retrospective cohort study about risk factors for late intestinal toxicity after radiotherapy, which may be included in this review. The other authors declare no competing interests for this review.

## Authors’ contributions

LW is responsible for the generation of the research question and design of the protocol, and will lead and oversee the study. QQ assisted with the generation of the research question and design of the protocol, and drafted the manuscript. QZ and QH assisted with the design of protocol, and QH provided guidance for developing the search strategy. XF and DC helped draft the manuscript and provided comments. All authors read and approved the final manuscript.

## Supplementary Material

Additional file 1 PRISMA 2009 Checklist and MOOSE Checklist. Click here for file

Additional file 2 MEDLINE Search Strategy. Click here for file

Additional file 3 Data Extraction Form (Cohort Study). Click here for file
