# Supplementary material for: Clinical risk factors for late intestinal toxicity after radiotherapy: a systematic review protocol
Source: Syst Rev. 2013 Jun 7;2:39. doi: 10.1186/2046-4053-2-39 (PMC3680145; doi:10.1186/2046-4053-2-39)
Supplement: Additional file 1 — PRISMA 2009 Checklist and MOOSE Checklist. [file 2046-4053-2-39-S1.pdf]

PRISMA 2009 Checklist

Section/topic	#	Checklist item	Reported on page #
**TITLE**			
Title	1	Identify the report as a systematic review, meta-analysis, or both.	1
**ABSTRACT**			
Structured summary	2	Provide a structured summary including, as applicable: background; objectives; data sources; study eligibility criteria, participants, and interventions; study appraisal and synthesis methods; results; limitations; conclusions and implications of key findings; systematic review registration number.	2
**INTRODUCTION**			
Rationale	3	Describe the rationale for the review in the context of what is already known.	3-4
Objectives	4	Provide an explicit statement of questions being addressed with reference to participants, interventions, comparisons, outcomes, and study design (PICOS).	4
**METHODS**			
Protocol and registration	5	Indicate if a review protocol exists, if and where it can be accessed (e.g., Web address), and, if available, provide registration information including registration number.	4
Eligibility criteria	6	Specify study characteristics (e.g., PICOS, length of follow-up) and report characteristics (e.g., years considered, language, publication status) used as criteria for eligibility, giving rationale.	5-6
Information sources	7	Describe all information sources (e.g., databases with dates of coverage, contact with study authors to identify additional studies) in the search and date last searched.	4
Search	8	Present full electronic search strategy for at least one database, including any limits used, such that it could be repeated.	4, Additional file 2
Study selection	9	State the process for selecting studies (i.e., screening, eligibility, included in systematic review, and, if applicable, included in the meta-analysis).	5-6
Data collection process	10	Describe method of data extraction from reports (e.g., piloted forms, independently, in duplicate) and any processes for obtaining and confirming data from investigators.	6
Data items	11	List and define all variables for which data were sought (e.g., PICOS, funding sources) and any assumptions and simplifications made.	6, Additional file 3
Risk of bias in individual studies	12	Describe methods used for assessing risk of bias of individual studies (including specification of whether this was done at the study or outcome level), and how this information is to be used in any data synthesis.	6-7
Summary measures	13	State the principal summary measures (e.g., risk ratio, difference in means).	7
Synthesis of results	14	Describe the methods of handling data and combining results of studies, if done, including measures of consistency (e.g., I^2) for each meta-analysis.	7

PRISMA 2009 Checklist

Section/topic	#	Checklist item	Reported on page #
Risk of bias across studies	15	Specify any assessment of risk of bias that may affect the cumulative evidence (e.g., publication bias, selective reporting within studies).	8
Additional analyses	16	Describe methods of additional analyses (e.g., sensitivity or subgroup analyses, meta-regression), if done, indicating which were pre-specified.	7
**RESULTS**			
Study selection	17	Give numbers of studies screened, assessed for eligibility, and included in the review, with reasons for exclusions at each stage, ideally with a flow diagram.	N/A
Study characteristics	18	For each study, present characteristics for which data were extracted (e.g., study size, PICOS, follow-up period) and provide the citations.	N/A
Risk of bias within studies	19	Present data on risk of bias of each study and, if available, any outcome level assessment (see item 12).	N/A
Results of individual studies	20	For all outcomes considered (benefits or harms), present, for each study: (a) simple summary data for each intervention group (b) effect estimates and confidence intervals, ideally with a forest plot.	N/A
Synthesis of results	21	Present results of each meta-analysis done, including confidence intervals and measures of consistency.	N/A
Risk of bias across studies	22	Present results of any assessment of risk of bias across studies (see Item 15).	N/A
Additional analysis	23	Give results of additional analyses, if done (e.g., sensitivity or subgroup analyses, meta-regression [see Item 16]).	N/A
**DISCUSSION**			
Summary of evidence	24	Summarize the main findings including the strength of evidence for each main outcome; consider their relevance to key groups (e.g., healthcare providers, users, and policy makers).	N/A
Limitations	25	Discuss limitations at study and outcome level (e.g., risk of bias), and at review-level (e.g., incomplete retrieval of identified research, reporting bias).	N/A
Conclusions	26	Provide a general interpretation of the results in the context of other evidence, and implications for future research.	N/A
**FUNDING**			
Funding	27	Describe sources of funding for the systematic review and other support (e.g., supply of data); role of funders for the systematic review.	9

From: Moher D, Liberati A, Tetzlaff J, Altman DG, The PRISMA Group (2009). Preferred Reporting Items for Systematic Reviews and Meta-Analyses: The PRISMA Statement. PLoS Med 6(6): e1000097. doi:10.1371/journal.pmed1000097

For more information, visit: **www.prisma-statement.org**.

MOOSE Checklist

	Reported on page	Brief Description
**Reporting of background should include**		
Problem definition	3	Moderate and severe late intestinal toxicity after radiotherapy causes significant damages. Identifying clinical risk factors is important for establishing predictive model and designing prevention strategies.
Hypothesis statement	4	What clinical factors increase the risk of moderate and severe late intestinal toxicity after radiotherapy for abdominal or pelvic malignancy.
Description of study outcomes	3	Moderate and severe late intestinal toxicity after radiotherapy
Type of exposure or intervention used	3-4	Clinical risk factors
Type of study designs used	5	Observational studies of cohort and case-control designs, randomized controlled trials and controlled clinical trials for interventions.
Study population	4	Patients that received radiotherapy for pelvic or abdominal malignancy.
**Reporting of search strategy should include**		
Qualifications of searchers (eg librarians and investigators)	4	Librarian and investigators with experience in systematic review.
Search strategy, including time period used in the synthesis and key words	4, Additional file 2	Structured terms of MeSH and free keywords will be used, which is detailed in Methods and Additional file 2.
Effort to include all available studies, including contact with authors	6	For studies without sufficient information to evaluate the eligibility, we will contact the study authors via emails to obtain their clarifications.

Databases and registries searched	4	MEDLINE, EMBASE, Web of Science, Cochrane library, Scoups, Google Scholar and Chinese BioMed.
Search software used, name and version, including special features used (eg explosion)	5	EndnoteX4 will be used to sort and screen studies. No other search software will be used.
Use of hand searching (eg reference lists of obtained articles)	4	Hand-searching will be used for the bibliography of retrieved studies and recent reviews.
List of citations located and those excluded, including justification	6	The search process will be presented in a flow chart. Studies excluded and justifications will be documented in the published review.
Method of addressing articles published in languages other than English	6	The abstracts or full-text of studies that are in the language other than English will be translated by Google Translate, and if the information is unclear, we will refer to the professional translation.
Method of handling abstracts and unpublished studies	6	For conference abstracts and other studies with missing data for synthesis or assessment of study quality, we will attempt to contact the study authors via emails, two times at least.
Description of any contact with authors	6	See above.
**Reporting of methods should include**		
Description of relevance or appropriateness of studies assembled for assessing the hypothesis to be tested	4-5	Detailed eligibility criteria are described in Methods.
Rationale for the selection and coding of data (eg sound clinical principles or convenience)	6	Data extracted is in accordance with study design, study characteristics, study population, and results.
Documentation of how data were classified and coded (eg multiple raters, blinding and interrater reliability)	6, Additional file 3	There are specific forms for cohort study, case-control study, and RCTs or CCTs.

Assessment of confounding (eg comparability of cases and controls in studies where appropriate)	7	Newcastle-Ottawa scale will be used for cohort studies and case-control studies. Cochrane Collaboration's tool for assessing risk of bias for RCTs will be used for studies of interventions.
Assessment of study quality, including blinding of quality assessors, stratification or regression on possible predictors of study results	7	Two reviewers will assess independently. Disagreement will be resolved by discussion or arbitration if necessary. Sensitivity analysis will be used.
Assessment of heterogeneity	7	Heterogeneity between included studies will be assessed by Cochran Q test and I^2 statistic.
Description of statistical methods (eg complete description of fixed or random effects models, justification of whether the chosen models account for predictors of study results, dose-response models, or cumulative meta-analysis) in sufficient detail to be replicated	7	The random effects model of Dersimonian and Laird will be used to pool the overall effect estimate, accounting for our assumption that there is potential variation among studies of different population, designs, primary malignancy, and radiation plans. Subgroup and sensitivity analysis will also be used.
Provision of appropriate tables and graphics	N/A	
**Reporting of results should include**		
Graphic summarizing individual study estimates and overall estimate	N/A	
Table giving descriptive information for each study included	N/A	
Results of sensitivity testing (eg subgroup analysis)	N/A	
Indication of statistical uncertainty of findings	N/A	
**Reporting of discussion should include**		
Quantitative assessment of bias (eg	N/A	

publication bias)		
Justification for exclusion (eg exclusion of non-English language citations)	N/A	
Assessment of quality of included studies	N/A	
**Reporting of conclusions should include**		
Consideration of alternative explanations for observed results	N/A	
Generalization of the conclusions (eg appropriate for the data presented and within the domain of the literature review)	N/A	
Guidelines for future research	N/A	
Disclosure of funding source	9	There is no external source of funding for this review.

From: Donna F. Stroup, PhD, MSc; Jesse A. Berlin, ScD; Sally C. Morton, PhD; Ingram Olkin, PhD; G. David Williamson, PhD; Drummond Rennie, MD; David Moher, MSc; Betsy J. Becker, PhD; Theresa Ann Sipe, PhD; Stephen B. Thacker, MD, MSc; for the Meta-analysis Of Observational Studies in Epidemiology (MOOSE) Group. Meta-analysis of Observational Studies in Epidemiology. A Proposal for Reporting JAMA. 2000;283(15):2008-2012. doi: 10.1001/jama.283.15.2008
