# Supplementary material for: Clinical risk factors for late intestinal toxicity after radiotherapy: a systematic review protocol
Source: Syst Rev. 2013 Jun 7;2:39. doi: 10.1186/2046-4053-2-39 (PMC3680145; doi:10.1186/2046-4053-2-39)
Supplement: Additional file 2 — MEDLINE Search Strategy. [file 2046-4053-2-39-S2.pdf]

MEDLINE Search Strategy

1. Radiation Injuries [MeSH]
2. Radiotherapy/ adverse effects [MeSH]
3. Radiotherapy/ mortality [MeSH]
4. Radiotherapy/ complications [MeSH]
5. radiotherapy [TW]
6. radiation [TW]
7. injur* [TW]
8. toxicit* [TW]
9. morbidit* [TW]
10. Intestinal Diseases [MeSH]
11. Gastroenteritis [MeSH]
12. Intestines [MeSH]
13. Lower Gastrointestinal Tract [MeSH]
14. intestine* [TW]
15. bowel [TW]
16. rectum [TW]
17. *rectal [TW]
18. Abdominal Neoplasms/ radiotherapy [MeSH]
19. Pelvic Neoplasms/ radiotherapy [MeSH]
20. Urogenital Neoplasms/ radiotherapy [MeSH]
21. Intestinal Neoplasms/ radiotherapy [MeSH]
22. Pancreatic Neoplasms/ radiotherapy [MeSH]
23. Animals [MeSH]
24. Humans [MeSH]
25. Review [Publication Type]
26. 7 or 8 or 9
27. 5 and 26
28. 6 and 26
29. 1 or 2 or 3 or 4 or 27 or 28
30. 12 or 13 or 14 or 15 or 16 or 17
31. 30 and 26
32. 10 or 11 or 31
33. 18 or 19 or 20 or 21 or 22
34. (23 not 24) or 25
35. 29 and 32 and 33
36. 35 not 34
