# Supplementary material for: Clinical risk factors for late intestinal toxicity after radiotherapy: a systematic review protocol
Source: Syst Rev. 2013 Jun 7;2:39. doi: 10.1186/2046-4053-2-39 (PMC3680145; doi:10.1186/2046-4053-2-39)
Supplement: Additional file 3 — Data Extraction Form (Cohort Study). [file 2046-4053-2-39-S3.pdf]

Data Extraction Form (Cohort Study)

Reviewer: *Date:*

Title:

First Author: Year of Publication:

Journal:

Funding Source:

Conflict of Interest:

Trial Code Designation:

Methods

Study Design: Prospective ☐/ Retrospective ☐

Study Period: Sample Size:

Study Population: Race/ Ethnicity:

Gender Ratio: Patients Age Range:

Exposure:

Control Condition:

Radiation Instrument: IMRT ☐/ 3D-CRT ☐/ Conventional ☐/ Others ☐

Radiotherapy Total Dose:

External Beam Radiation Dose: Brachytherapy Dose:

Dose Fractionation: Target Area Arrangement:

Results

Primary Malignancy: No. of Cases:

Follow-up Period: No. of Patients Lost to Follow-up:

Endpoint Definition:

Risk factor:

Crude RR: CI: SE:

Adjusted RR: CI: SE:

Notes:
